# Beyond the Dura ― Massive Hyperostotic Response from a Convexity Meningioma: Systematic Review & Illustrative Case Example

**DOI:** 10.1055/a-2967-3259

**Published:** 2026-10-12

**Authors:** MacKenzie Steven Thomas, MacGregor C. Thomas, Kari O'Toole, Mark M. Mims, Christopher Salvatore Graffeo

**Affiliations:** 1 Department of Neurosurgery 6186 The University of Oklahoma Health Sciences CenterOklahoma CityOklahomaUnited States; 2 Department of Otolaryngology-Head and Neck Surgery 6186 The University of Oklahoma Health Sciences CenterOklahoma CityOklahomaUnited States

**Keywords:** hyperostosis, meningioma, calvarium, TRAF7, convexity meningioma, calvarial hyperostosis

## Abstract

**Introduction** Meningiomas account for approximately 41% of primary CNS neoplasms, a minority of which are associated with paracrine-induced cranial hyperostosis. We report a unique systematic review of this rare phenomenon alongside an illustrative case of a woman with 14 years of progressive pan-calvarial hyperostosis attributable to a bilateral parasagittal meningioma.

**Methods** A novel case of massive cranial hyperostosis treated surgically and an associated PRISMA-compliant systematic review are presented. Searches of OVID MEDLINE and EMBASE (keywords “Hyperostosis” and “Meningioma”) on April 2, 2025 yielded 384 sources. All studies reporting calvarial hyperostosis associated with a convexity meningioma were included. Studies prior to the year 2000, non-human, or those that reported hyperostosis in other skull-base regions were excluded.

**Results** A woman in her mid-40s presented with more than 14 years of enlarging pan-calvarial skull deformity predominantly involving the bilateral frontal bones. MRI revealed an enhancing midline mass obliterating the superior sagittal sinus consistent with meningioma. She underwent bifrontal craniotomy with *en bloc* hyperostotic bone removal and microsurgical meningioma resection. Reconstruction used dural grafting, titanium mesh cranioplasty, and scalp excision. Pathology confirmed a meningothelial meningioma with brain invasion (WHO Grade 2) with a solitary *TRAF7* mutation. Systematic review yielded 25 studies (107 patients) showing a high prevalence of bifrontal bony involvement and meningothelial histology in cranial hyperostosis.

**Conclusion** Mutations in *TRAF7* , a regulator of NF-κB, MAPK, and related pathways, are linked to calvarial hyperostosis and meningothelial histopathology. This systematic review and uniquely severe case highlight the potentially profound impact of *TRAF7* mutation on paracrine hyperostosis.

## Introduction

Meningiomas account for approximately 40% of primary intracranial neoplasms in the United States, or an incidence rate of 9.73 per 100,000 person-years. 1 Predominantly benign, meningiomas arise from the dural lining of the neuroaxis and demonstrate phenotypically distinct behaviors derived in part from their neuroanatomic origin. Calvarial dura is the most common source for intracranial meningiomas, accounting for a substantial majority of incident cases. 2

Meningiomas have the potential to impart mass effect on the underlying neural tissues, which may prompt neurosurgical treatment. Infrequently, mass effect arises not from the meningioma itself, but from an induced paracrine hyperostotic expansion of the overlying bone, thought to be attributable to meningiomatous cell invasion of Haversian canals precipitating osteoblast stimulation and bone growth. 3 Hyperostosis occurs in 4.5% of meningiomas, many of which demonstrate *en plaque* morphology and are located in the spheno-orbital region. 4 5 We report a unique case of a bilateral parasagittal meningioma inducing massive bilateral frontoparietal convexity hyperostosis, together with a contextualizing literature review.

## Case Report

### Initial Presentation

A 42-year-old woman presented with bilateral frontal skull enlargement, reportedly progressive over “at least 14 years.” Apart from cosmetic changes, occasional headache, and right-sided aural fullness worsening over several months, she remained neurologically intact. Brain MRI demonstrated a broad-based bilateral contrast-enhancing parafalcine mass lesion extending from the glabella anteriorly to the post-central gyrus posteriorly, highly consistent with a meningioma ( Fig. 1A–D ). Additional lesions consistent with meningioma were noted in the right cavernous sinus extending into the pterygopalatine fossa and along the underside of the left lesser sphenoid wing. Multiple sequelae of intracranial hypertension were also noted. CT venography showed occlusion of the superior sagittal sinus anteriorly, with profound central stenosis toward the posterior mass, and subsequent reconstitution at the central sulcus. Given the large compressive mass with profound progressive bony changes ( Fig. 2A,B ), neurosurgical treatment was recommended.

**Fig. 1:**
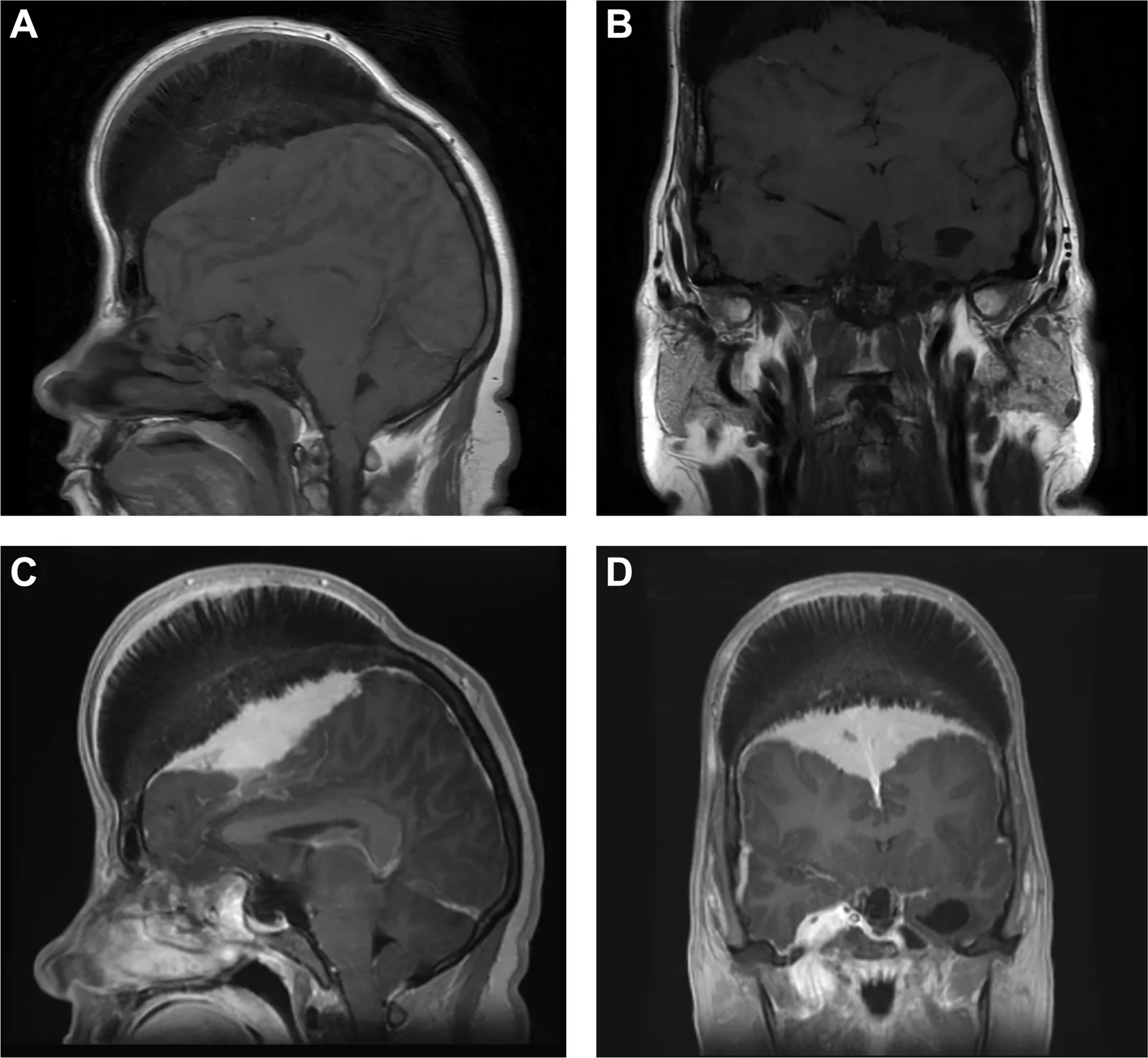
Preoperative magnetic resonance imaging of a hyperostosing parafalcine meningioma. **(A)** Sagittal T1-weighted image, **(B)** coronal T1-weighted image, **(C)** sagittal multiplanar reconstruction (MPR) post-contrast T1-weighted image, and **(D)** coronal multiplanar reconstruction (MPR) post-contrast T1-weighted image demonstrating a broad-based bilateral contrast-enhancing parafalcine mass lesion extending from the region of the glabella anteriorly to the post-central gyrus posteriorly, highly consistent with a meningioma.

**Fig. 2:**
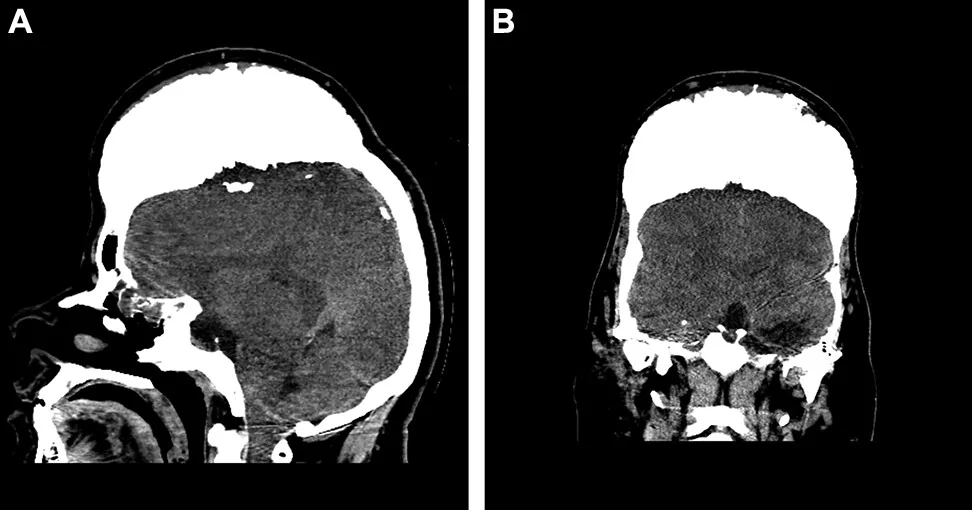
Preoperative computed tomography. **(A)** Sagittal and **(B)** coronal non-contrast CT bone-window images showing marked calvarial hyperostosis associated with the bilateral parasagittal meningioma.

### Intraoperative Course

Under anesthesia, a curvilinear bi-coronal incision was made with anticipation of scalp resection for contouring and optimal cosmesis. The scalp flap was raised, reflected anteriorly, and retained in place ( Fig. 3 ). Using multiple 6-mm cutting burrs, a circumferential trough was then fashioned just inferior to the hyperostotic bone. As the dura was exposed, a plane was developed circumferentially, allowing the inner table to be released from the calvarium and delivered *en bloc* for pathological analysis ( Fig. 4A,B ). Intraoperative sampling confirmed meningioma.

**Fig. 3:**
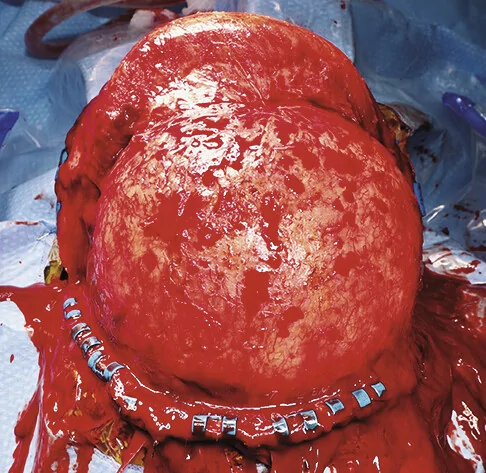
Intraoperative photograph of bi-coronal scalp flap exposing the hyperostotic calvarium.

**Fig. 4:**
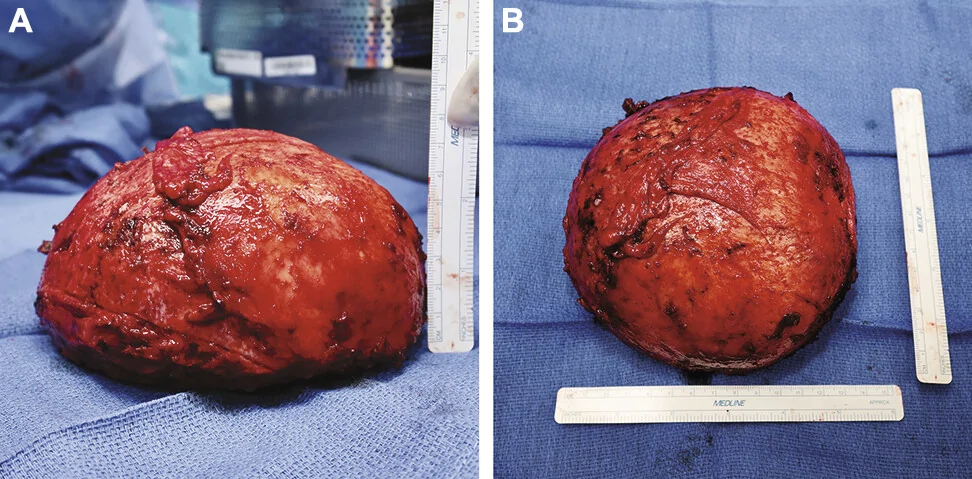
Resected calvarial bone flap from *en bloc* craniotomy. **(A)** Specimen measuring approximately 8 cm in height. **(B)** Specimen measuring approximately 14 cm × 15 cm.

The dura was opened and the tumor was extensively resected to the level of the falx while preserving the underlying cortex, superior sagittal sinus, and draining veins. The mass was debulked, leaving an adherent thin rind of tumor on the sinus. Doppler ultrasound confirmed anterior occlusion of the superior sagittal sinus by the tumor. At the posterior limit of the exposure, the superior sagittal sinus reconstituted, and several large draining veins were noted to enter the sinus at this level. Thereafter, the sinus was coagulated, ligated, and divided just anterior to the stenotic reconstitution, taking great care to preserve clusters of adjacent draining veins associated with the bilateral supplemental motor areas (SMAs). This allowed for a Simpson grade III near-total resection of the mass. 6 Following resection, the meninges were reconstructed with two large DuraGen grafts, and the calvarium was reconstructed with a pair of cranioplasty mesh plates ( Fig. 5 ). The facial plastics team then performed a subtotal scalp resection for recontouring, and the wound was closed in layers over multiple subgaleal drains.

**Fig. 5:**
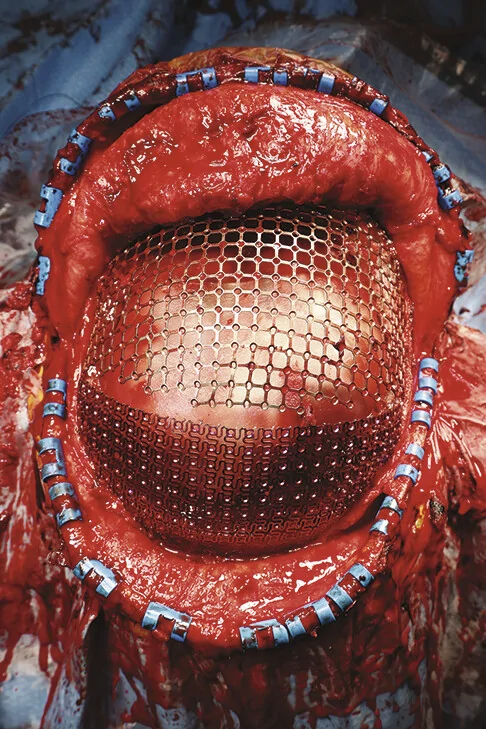
Intraoperative photograph showing mesh placement following bifrontal craniotomy and near-total tumor resection.

### Initial Postoperative Outcome and Late Follow-up

Postoperatively, the patient remained hemodynamically stable but awoke slowly. An immediate head CT was reassuring, with expected postoperative changes and no evidence of hematoma or other pathologic findings. She remained deeply lethargic after several hours, moving all extremities briskly but neither opening eyes nor responding to commands. MRI revealed new intraparenchymal infarcts with hemorrhage involving the bilateral SMAs, right greater than left ( Fig. 6A–C ). The patient had a prolonged hospitalization due to her comatose state and was transferred to a skilled facility followed by inpatient rehabilitation and ultimately home with her family. At 6-month postoperative evaluation, she was fully back to her cognitive baseline. Follow-up MRI showed no evidence of recurrent disease, stable postoperative changes with complete resection of the mass, and resolution of the subacute postoperative venous hemorrhages and associated parenchymal signal changes ( Fig. 7A,B ). Final pathology confirmed WHO Grade 2 meningothelial meningioma with brain invasion, including mutated tumor necrosis factor receptor-associated factor 7 ( *TRAF7* ). 7

**Fig. 6:**
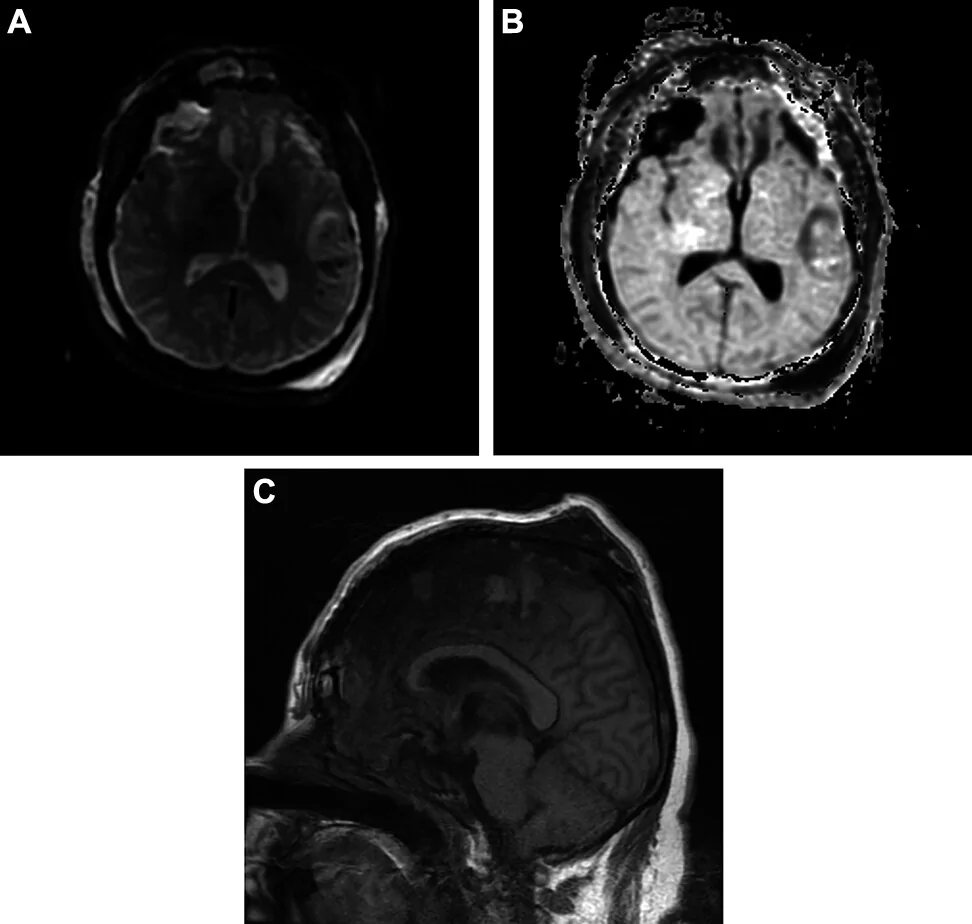
Postoperative magnetic resonance imaging showing supplemental motor area (SMA) infarcts with hemorrhage. **(A)** Axial diffusion-weighted imaging, **(B)** axial exponential apparent diffusion coefficient (ADC) image, and **(C)** sagittal T1 FLAIR.

**Fig. 7:**
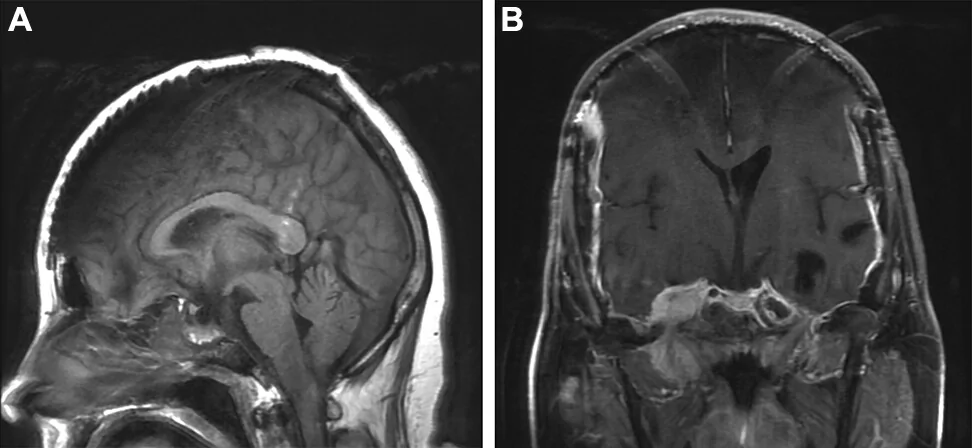
Magnetic resonance imaging at 6-month follow-up. **(A)** Sagittal and **(B)** coronal T1 FLAIR images confirming near-total resection with cranioplasty in place and no residual mass.

## Methods

A systematic review of the literature on meningioma-associated hyperostosis was conducted according to PRISMA 2020 guidelines. 8 OVID MEDLINE and EMBASE (Classic + EMBASE) databases were searched on April 2, 2025 (2000–2025) using the terms “hyperostosis” and “meningioma.” Studies reporting calvarial hyperostosis associated with convexity meningiomas were included; pre-2000, non-human, and skull-base cases were excluded. Titles and abstracts were screened independently by two reviewers (M.S.T., M.C.T.). Twenty-five studies met inclusion criteria ( Fig. 8 ; see **Supplemental Materials S1 and S2** (online only) for complete methods and PRISMA 2020 checklist).

**Fig. 8:**
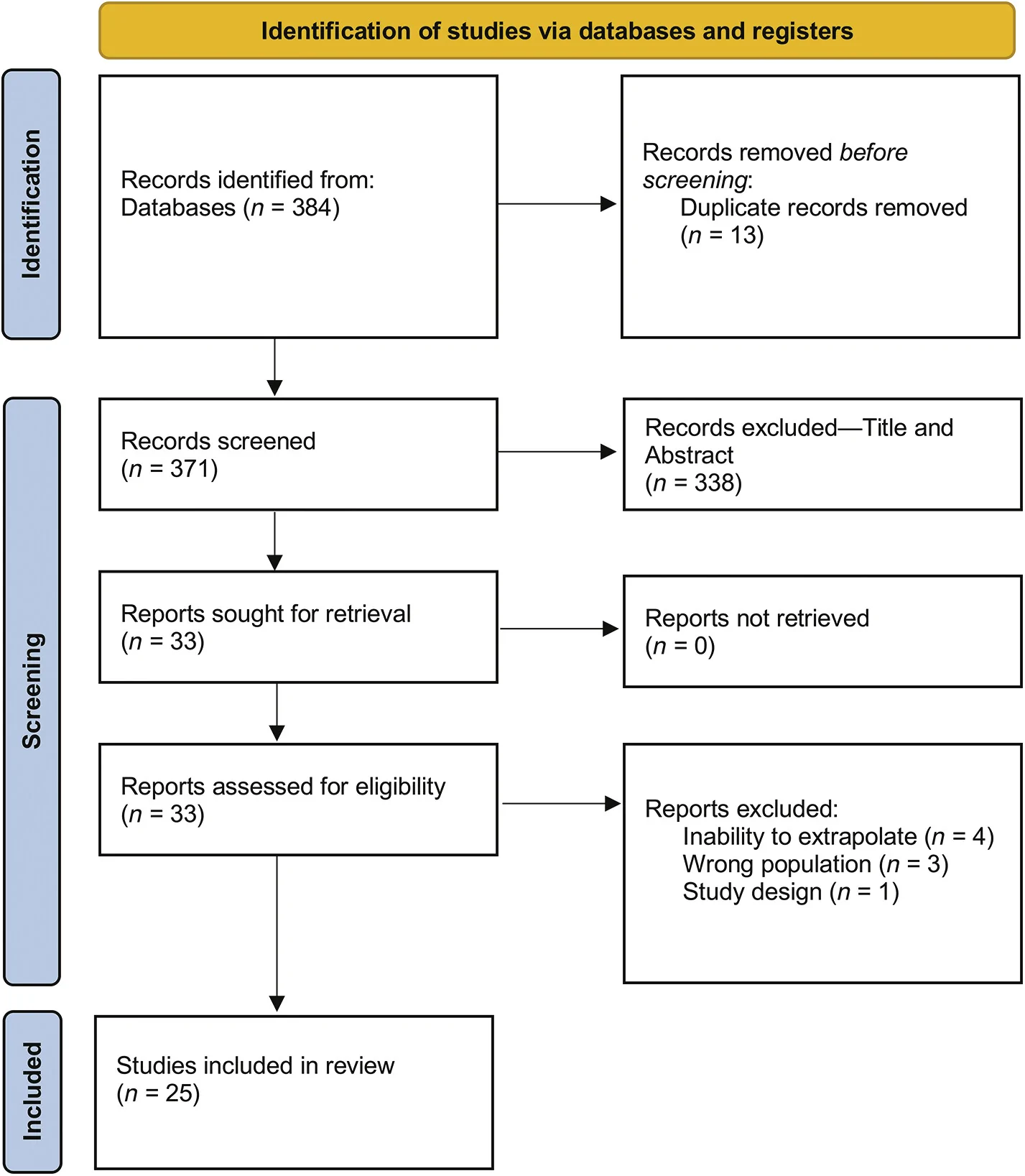
PRISMA flow diagram of the literature search and selection process for reports on hyperostosing cranial vault meningiomas.

## Results

Literature review yielded 25 articles, encompassing 107 patients—71 females and 36 males, with an average age of 48.59 years. 3 4 9 10 11 12 13 14 15 16 17 18 19 20 21 22 23 24 25 26 27 28 29 30 31 Calvarial hyperostosis was most frequently observed in the frontal region (32.7%), followed by the parietal (28.0%), frontoparietal (22.4%), parieto-occipital (4.7%), temporal (3.7%), frontotemporal (2.8%), temporal-parietal (1.9%), occipital (0.9%), frontoparietotemporal (0.9%), frontoparietooccipital (0.9%), and bi-coronal midline (0.9%) regions. Most patients demonstrated focal hyperostosis over regions ranging from 1.5 to 10.7 cm in diameter, with only 10 cases with similar pan-calvarial changes to those noted in the present case. 9 13 14 16 17 19 24 25 30 31

## Discussion

We report a novel case of massive cranial hyperostosis, progressive over 14 years, treated surgically and complicated by subacute bilateral SMA venous infarcts precipitating a prolonged but ultimately complete neurological recovery, with an associated literature review. Although mild hyperostotic changes are an uncommon but well-described occurrence in meningioma, it predominantly involves the spheno-orbital complex and is relatively mild where noted. Massive calvarial hyperostosis is exceedingly uncommon, with no more than 11 cases described in the neurosurgical literature, including the present report.

The current and preceding cases share several features, including frontal bone–centered morphology and meningothelial histopathology. This subtype was observed in 8 of 10 comparable cases, while the remaining two demonstrated lipomatous and chordoid variants. 9 13 14 16 17 19 24 25 30 31 After excluding 18 patients without reported histology, 67% of all patients with calvarial meningioma-associated hyperostosis also demonstrated meningothelial histopathology. 3 4 10 11 12 15 18 20 21 22 23 26 27 28 29 Interestingly, a study analyzing 3,000 meningiomas found that a sole *TRAF7* mutation is moderately associated with meningothelial histology (OR 2.19; *p* = 0.00468). 32 This highlights an important area for future study, namely, a more focused assessment of the interplay between histologic subtype, tumor grade, *TRAF7* mutation, and incidence of massive meningioma-associated calvarial hyperostosis.

The lone genetic abnormality of known clinical significance identified in our patient was a somatic mutation of *TRAF7* , which has been identified as a prominent driver of tumorigenesis in up to one in four meningiomas. 33 TRAF7 is an important regulator of several pathways, providing positive regulation of CHOP transcription factor, which in turn modulates the cellular stress response to genomic damage and misfolded proteins. 34 TRAF7 also has E3 ubiquitin ligase activity, enabling it to mark important genomic regulators for destruction, such as NF-κB essential modulator (NEMO), the anti-apoptotic c-FLIP, the p65 subunit of the transcription factor NF-κB, and the tumor suppressor p53. 35 Due to its wide range of effects and the possibility for additional as-of-yet-undiscovered functionalities, the mechanistic role of *TRAF7* mutation in meningioma is incompletely understood, and potentially polyfactorial, variably penetrant, or both.

The identification of a *TRAF7* mutation in the present case aligns with several key preceding findings exploring the genetic underpinnings of meningioma hyperostosis. Recent work by Jin et al. characterizing a small sample of sphenoid wing meningiomas identified a complex and potentially causal relationship between morphologic and anatomic patterns of bony overgrowth with mutation burden, specifically identifying a more than 4-fold risk of hyperostosis among meningiomas harboring *TRAF7* mutation, as compared with other benign, sporadic meningiomas lacking this abnormality. 36 Another cohort study analyzing 22 genes across a sample of 342 benign sporadic meningiomas found that *TRAF7* was the only genetic abnormality significantly associated with hyperostotic changes ( *c* ^2^ = 15, *p* = 0.000092). 37 The potentially polyfactorial nature of *TRAF7* mutation expression in meningioma is also highlighted by our patient, with attention to key distinctions from the meningioma population at large, such as the predominance of spheno-orbital and other skull base locations among *TRAF7-* mutated meningiomas in general, rather than along the calvarium. 1 32 35 36 37 This genotype-location discordance is notable, as convexity meningiomas historically arise from neurofibromatosis-2 ( *NF2* ) gene inactivation or chromosome 22q loss. 1 32 33 Genomic subgroup affects not only location but also prognosis. Work by Youngblood et al. analyzed 469 meningiomas and found that *TRAF7* -mutated tumors, along with Hedgehog-, *NF2-* , and PI3K-altered subgroups, demonstrate higher recurrence rates than *KLF4-* , *POLR2A-* , and *SMARCB1-* driven tumorigenesis. 38 The present case underscores the importance of close longitudinal surveillance, given the tumor’s genetic profile, brain invasion, and Simpson grade III resection. As noted, these unusual features emphasize the need for deeper and more nuanced mechanistic explorations of *TRAF7* mutation impacts on meningioma growth, development, and prognosis.

## Conclusion

Meningiomas with massive hyperostosis affecting the cerebral convexity are exceedingly rare. We report a unique case of a young woman who presented with more than 14 years of progressive calvarial hyperostosis induced by an underlying WHO Grade 2 meningothelial, *TRAF7-* mutated bilateral parasagittal meningioma, which was treated with *en bloc* calvarial craniectomy, Simpson grade III near-total tumor resection, titanium mesh cranioplasty, and scalp resection and reconstruction. Her postoperative course was initially complicated by subacute venous infarcts involving the bilateral SMAs, potentially secondary to the ligation of the superior sagittal sinus resulting in turbulent flow in the postoperative setting, from which she ultimately mounted a complete recovery. The present case highlights both an exceedingly rare clinical entity, as well as a critical area for future study, namely, the interplay between meningioma histology, genetics, anatomical location, and the development of hyperostotic changes.

## Data Availability

The underlying research data of this article is available in the Supplementary Material file section of the published article.

## Declaration of GenAI Use

Generative AI was used solely for grammar and language editing during manuscript preparation. The authors reviewed all suggested edits and made alterations as needed before incorporation.

## Supplementary Material

Ergänzendes Material Supplementary Material
